# An appraisal on enablers for enhancement of waste cooking oil-based biodiesel production facilities using the interpretative structural modeling approach

**DOI:** 10.1186/s13068-021-02061-2

**Published:** 2021-11-06

**Authors:** Rajendra Kukana, O. P. Jakhar

**Affiliations:** Engineering College Bikaner, Bikaner, Rajasthan India

**Keywords:** Waste cooking oil, Interpretative structural modeling, Biodiesel production, Multiple criteria decision-making, Enablers for promotion

## Abstract

With the continuous depletion of energy sources globally and serious concern regarding environmental degradation by the use of fossil fuel, biodiesel may play a key transponder. Biodiesel blended with diesel fuel achieves a decreased environmental footprint without losing the reliability of output and consumption. Biodiesel is produced from a variety of sources. Biodiesel generation from waste cooking oil (WCO) is effective for both the atmosphere and human health. Many research studies reported WCO biodiesel as a potential alternative fuel for internal combustion engine. The present study aims to provide key promoting and implementing agents for WCO utilization and WCO-based biodiesel production. A systematic literature review has been performed to identify enablers and the contextual relationship between various enablers was developed using interpretative structural modeling (ISM) and expert views. Using the method of ISM and cross-impact matrix multiplication applied to classification (MICMAC) methodology, the impact of enablers is studied. The findings revealed that all established enablers play an important role and are equally important promoters for the development of biodiesel based on WCO. The findings further suggest that human health issues, biodiesel processing plants, biodiesel support vehicles, and biodiesel production technology play a key role in the manufacture of biodiesel dependent on WCO. The most important leaders in the development of WCO biodiesel are government policy and funding, confidence in environmental issues, and financial assistance to biodiesel manufacturers.

## Introduction

Energy use is growing day by day due to the growth of the world population, the emergence of technological advances, and the development of living standards. Coal, oil, and gas will continue to dominate until at least 2050. The percentage of fossil fuels will fall to 77%, down from 79% in 2010. Renewable energy sources will contribute to around 20% of global energy consumption by 2050, up from approximately 15% in 2010. Nuclear energy will provide around 4% of total primary energy output in 2050, up from 6% in 2010.Global electricity generation will have grown from 123 to 150% by 2050 [1]. Global energy consumption is expected to rise by 4.6% in 2021, more than mitigating a 4% drop in 2020 and driving demand 0.5% higher than in 2019. Despite a projected yearly growth of 6.2% in 2021, global oil consumption is expected to be about 3% lower than in 2019.Even at end of 2021, oil usage for aviation is expected to be 20% lower than in 2019, with yearly demand being more than 30% lower than in 2019. As demand for coal, oil, and gas recovers with the economy, global energy-related CO_2_ emissions are expected to return and climb by 4.8% in 2021 [2]. Since carbon oil is widely used, it causes a slow depletion of the natural environment by Shrigiri et al. [3] and Joshi et al. [4]. The alternative fuel supply must be eco-friendly, thus acknowledging environmental concerns [5]. This prompted the researchers to recommend that biodiesel is the best option for all alternative fuels. Biodiesel, unlike other petroleum products, is a sustainable energy source that will be phased out over time [6]. It can be generated on demand and emits less pollution than petroleum diesel because it is derived from renewable resources [7]. One of the most significant advantages of utilizing biodiesel is that it can be used in conventional diesel engines with few or no changes, it is preferred as the primary transport energy source to replace fossil fuels [8]. Biodiesel can be used alone (B100) or in mixtures with petroleum diesel. Because it is almost sulfur-free, it improves engine lubrication and extends engine life [9]. It is safer to store, handle, and operate than industrial diesel because it is non-toxic, biodegradable, and has a high flash point [10]. When fossil fuels are burned, greenhouse gases such as carbon dioxide are released into the atmosphere, raising the temperature and causing global warming [11]. Biodiesel, which is made from indigenous energy sources, can serve as an alternate fuel source and lessen our reliance on foreign oil sources. It is made at local refineries, the dependency on imported petroleum is minimized by biodiesel [12]. When biofuels are burned, it ejects much less carbon dioxide and creates very few pollutants. Biodiesel generates less soot (particulate matter), carbon monoxide, unburned hydrocarbons, and sulfur dioxide than petroleum diesel [13]. It would be possible to minimize the quantity of oil imported into a nation if countries could start generating biodiesel on their own. As a result, the country’s economy might be balanced, and geopolitical tensions could be reduced. Renewability, oxygen content and greater lubrication are the main benefits among others [14]. Cooking is done using edible oils all over the world, and the waste of that oil is discarded. The waste cooking oil (WCO) poses significant challenges for society in terms of environmental, societal, economic, and health [15–17]. The terminology "waste cooking oil" (WCO) refers to vegetable oil that has been used in the preparation of food, but is no longer suitable for use in food manufacturing [18]. WCO can come from a variety of places, including the home, business, and industry. WCO is a potentially hazardous waste stream that needs careful treatment [19–21]. Further, at present, there is the penalty of the organized method devised for the collection of household waste cooking oils. Water pollution results if the amount of WCO is dumped through the drain [22]. The drainage of waste cooking oil into water sources increases the number of chemical compounds in water. This has a direct impact on water safety and, as a result, on the lives of fish populations, other marine life, and the local environment [23]. Since it recycles WCO and offers low-pollution green fuels, biodiesel processing from WCO is environmentally sustainable [24]. WCO is two to three times less expensive than vegetable oils, and it also saves money on waste removal and treatment Jacobson et al. [25]. Meanwhile, the amount of farmland necessary for biodiesel-producing crops is significantly reducing [26]. It lowers the expense of waste disposal while also replacing some petrochemical oil imports. The economic, environmental, and waste management aspects of biodiesel production from used cooking oil are all important [27, 28]. The hydrolysis of triglycerides and the percentage of free fatty acid in the oil are increased during cooking due to the heating and water addition. The transesterification reaction is slowed down by the presence of water and free fatty acid (FFA) [29, 30]. Biochar is also widely used for biodiesel production and is gaining popularity due to its heterogeneous nature; various methods are used to activate biochar, and novel activation approaches are being developed to make biochar accelerators more productive and recyclable [31–35]. Heterogeneous catalyst biochar produced from pyrolysis of cork at 600 °C in 6 h with 98% FAME conversion [32]. Lipases are used for enzymatic transesterification, and their use was limited due to enzyme inactivation by insoluble methanol droplets, resulting in a lower fatty acid methyl ester (FAME) yield [11]. Homogeneous alkali catalysts are used in the majority of industrial operations (e.g., NaOH or KOH) [9, 30]. Compression ignition (CI) engine operating with WCO biodiesel has the ability to fulfilling standards of emission as per requirements published by Phan and Phan [36]. Biodiesel consists of 10–12% of oxygen by weight which enables complete combustion and produces less emission. Using waste cooking oil biodiesel and its blends with diesel, a reduction of CO and HC emissions from engines was achieved [37]. Besides all the advantages and benefits of WCO, biodiesel from WCO is still not produced by industry at a mass scale. At this stage to promote the recycling of WCO, there is an urgent need to study the key factors responsible for the promotion and leading agents. To encourage the production and promotion of the WCO biodiesel enabler’s analysis is important. Many researchers have used the ISM technique for the analysis of enabler model analysis the technique has been used for the solution of complex problems in the management of decision-making. ISM (interpretive structural modeling) is a well-known technique for determining the relationships between particular objects that define an issue [38, 39]. A collection of separate explicitly and indirectly connected components is organized into a rigorous systematic model using this methodology [40–42]. The model depicts the layout of a dynamic issue or problem in a well-designed pattern that includes both graphics and words presented by Jacob et al. [44]. A variety of variables can be relevant to an issue or concern in any complex problem under consideration. They established an interrelationship among supply risk factors based on the interdependence and driving force of the various factors. Their research aided supply chain risk managers in making resource management decisions [44–46]. It is observed from the literature that a little attempt has been made, to the authors’ knowledge, to identify the essential enablers in biodiesel production from WCO biodiesel. The research is lacking in terms of aspects hindering the application of factors impacting biodiesel production and their contextual connections. To fill this research gap, this study presents empirical evidence to improve knowledge of factors that promote biodiesel production, which will benefit biodiesel producers, WCO suppliers, and environments. In the present research investigation enablers for waste cooking biodiesel in India have been identified through literature and expertise in automobile and biodiesel.

### Problem formulation

To overcome issues and promote the production facilities of waste cooking oil biodiesel, we have identified enablers for a large-scale production facility in India. These enablers are identified by literature (mentioned in Table 1) and with the help of experts from the field of industry and academic opinion in interviews.

**Table 1:** Formulation of enablers for the processing of biodiesel from waste cooking oil

No.	Enablers	Comments	Sources
1	Awareness to used cooking oil as biodiesel	Used cooking oil drained or repetitive use, main culprit	[58, 62, 66]
2	Human health concern	Used cooking oil misleading human health since is it used as edible oil	[52, 58]
3	Used cooking oil collection center	The collection centers are most influenced by biodiesel production using waste/used cooking oil. To enhance biodiesel production, a sufficient number of collection centers must be established	[54–57]
4	Biodiesel production facility	To meet the demand for alternating sources of fossil fuel large-scale production facility for biodiesel required	[50, 62, 66]
5	Adequate supply of used cooking oil	Having a steady supply of waste cooking oil for a large-scale manufacturing plant is like breathing fresh air	[53, 60]
6	Production Technology for biodiesel	To meet emission standard and engine compatibility UCO/WCO biodiesel production technology play a vital role	[50, 51, 53]
7	Belief in environment concern	WCO/UCO affects the environment adversely and WCO biodiesel supportive to nature to diesel fuel	[52, 61–64]
8	Availability of technical expertise	To utilized the WCO and for research and development, an expert in the field required	[59, 64]
9	Financial assistant to biodiesel producer	To promote the production of WCO-based biodiesel financial support Tax, etc.	[59, 65]
10	Quality of human resources	Technical manpower and their training	[59, 64]
11	Biodiesel supportive vehicles	Creating a market for biodiesel production, promoting the people for WCO biodiesel	[56, 61]
12	Government policy and support	Incentives and benefits offered by the government for production and use	[55, 63, 65]

### Analysis using ISM approach

**Fig. 1:**
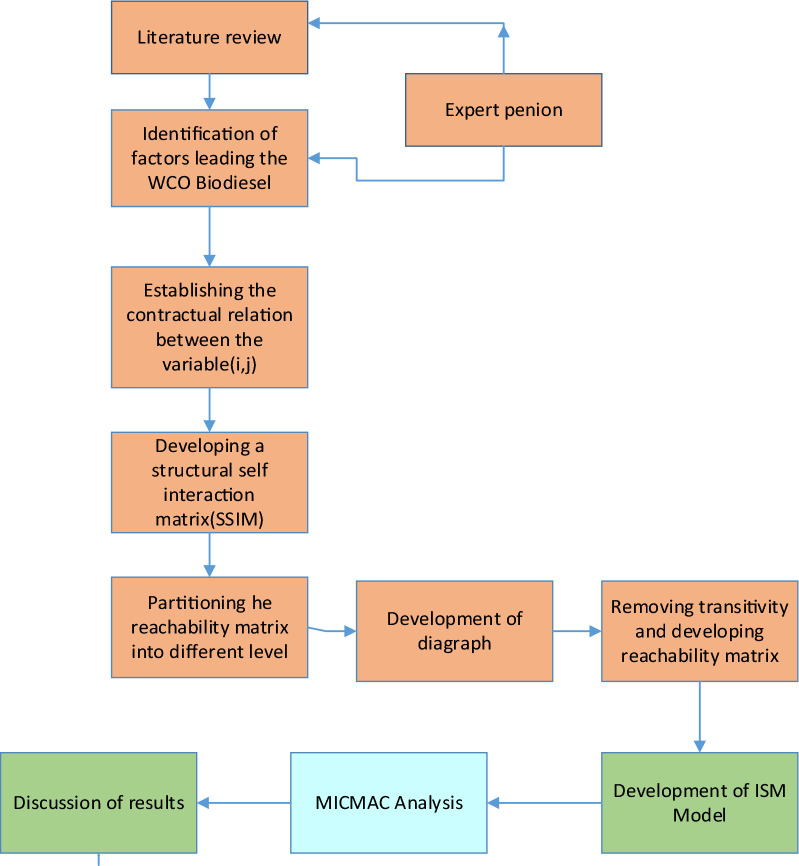
ISM methodology

In a complex system, some techniques, such as Analytical Network Process (ANP) and ISM, are available to analyze the interrelationships between variables. The ISM approach is suggested in the current research review framework to analyze the relationship between identified enablers. ISM transforms ambiguous and complex structures into well-defined models and portrays [38, 39]. The situation is much more reliable than the individual factor taken into isolation, defined directly and indirectly among the factors [40]. The schematic flow of the methodology is shown in Fig. 1. ISM begins with the selection of variables that are important for analysis and then progresses with the methodology of analyzing a group problem. It is a type of modeling, where a digraph model represents the basic relationships and overall structure reported by Jacob et al. [43].

The steps to be taken for ISM technique evaluation are as follows:

#### Stage I: elements recognition

Determine the WCO biodiesel production enablers whose interrelationships are identified.

#### Stage II: describing contextual connections

The qualitative relationship between the factors listed is made up of experts from the area of interest.

#### Stage III: interpreting relationships

The contextual relationships are defined by ISM. Working with these established contextual relationships is also essential.

#### Stage IV: development of the matrix of structural self-interaction

This ISM step involves the creation of a self-interaction structure Matrix (SSIM) by comparing variables correlated in step III in a pair-wise manner. The input for this step was the contextual relationship developed in step III, and the existence of two sub-elements (i and j) along with their associated direction was discussed. In this work, binary is considered as (i, j) matrix only.

#### Stage V: matrix of reachability and verification of transitivity

The SSIM developed in step III has been converted into an accessibility modeling that matrix, the reachability matrix (RM), and transitivity were examined. This indicates that if variable A is associated with variable B, and if variable B is associated with variable C, variable A is also associated with variable C. In terms of entries i and j, there is no direct or indirect relationship from i to j if i, j of an RM is 0.

#### Stage VI: partition level on the matrix of reachability

A level partition procedure is executed to acknowledge the levels of the specified elements across two sets, i.e., the accessibility set and the antecedent set. This will demonstrate that, in the case of elements at a specific level, the intersection of the accessibility set and the antecedents set matches the accessibility setting. First, from the set of elements, the top-level elements satisfying the aforementioned condition should be excluded and the procedure is performed until all ISM model levels have been established.

#### Stage VII: the diagraph is created

At specified levels, the elements are graphically ordered and the links are indicated according to the relationship shown in the accessibility matrix. By eliminating transitivity, a diagraph is derived.

#### Stage VIII: model creation

By replacing the nodes in the diagraph with boxes containing components, the diagraph is transformed into an ISM model.

### Matrix of systemic self-interaction (SSIM)

In establishing the association among the enablers, ISM suggests using the opinions of experts. Therefore, experts from the biodiesel development industry and academics were consulted for the analysis to identify the relevant connections among the enablers for WCO biodiesel production. A contextual relationship of ‘leads to’ was defined for evaluating the variables. That means another factor is affected by one factor. Based on this, a conceptual relationship was formed between the factors defined. The related trajectory of the relationship was challenged, taking into account the qualitative relationship of each factor and the nature of a relationship between two factors *i* and *j*.

The following notations as below considered for indicating the interaction path between two variables *i* and *j*:

- *I* = parameter *i* is going to contribute to factor *j*
- *J* = parameter *j* is caused by factor *i*
- *B* = parameter *i* and *j* relate to each other
- *N* = element *i* and *j* do not add to one another.

SSIM was developed for WCO biodiesel production based on the contextual relationship shown in Table 2. The association of 12 enablers for the processing of waste cooking oil is shown in Table 2 which reveals that knowledge of WCO as biodiesel supports the concern for human health while representing the partnership of ‘J’ as enablers of SSIM. Appropriate production enablers encourage the use of vehicles promoting biodiesel. Therefore, for the relation with these enablers, ‘I’ is represented in the SSIM. Government policy and enabler funding encourage environmental conviction. Accordingly, ‘B’ will describe the relationship between these enablers in the SSIM. There is no connection between the supply of used cooking oil and the enablers of biodiesel support vehicles, so the link between these enablers is expressed in the matrix by ‘N’.

**Table 2:** Matrix of systemic self-interaction

	Enablers	**12**	**11**	**10**	**9**	**8**	**7**	**6**	**5**	**4**	**3**	**2**	**1**
1	Awareness to used cooking oil as biodiesel	J	I	I	J	J	J	I	I	I	I	I	
2	Human health concern	J	N	J	J	J	J	J	J	I	J		
3	Used cooking oil collection centers	J	N	J	J	N	J	J	I	I			
4	Biodiesel production facility	J	I	I	J	I	J	J	J				
5	Supply of used cooking oil	J	N	N	J	J	J	N					
6	Production Technology for biodiesel	J	J	J	N	J	J						
7	Belief in environment concern	B	I	I	I	N							
8	Availability of technical expertise	J	I	I	J								
9	Financial assistant to biodiesel producer	J	I	I									
10	Quality of human resources	J	I										
11	Biodiesel supportive vehicles	J											
12	Government policy and support												

### Matrix for initial and final reachability

The SSIM needs to be transformed into a binary matrix in this process. The next step in the ISM method is to create an initial SSIM usability as shown in Table 3. To this end, SSIM is converted into an initial accessibility matrix by substituting 1 s or 0 s in the initial accessibility matrix for the symbols I, J, B, or N of SSIM.

**Table 3:** Initial reachability matrix

	Enablers	**1**	**2**	**3**	**4**	**5**	**6**	**7**	**8**	**9**	**10**	**11**	**12**
1	Awareness to used cooking oil as biodiesel	1	1	1	1	1	1	0	0	0	1	1	0
2	Human health concern	0	1	0	1	0	0	0	0	0	0	0	0
3	Used cooking oil collection center	0	1	1	1	1	0	0	0	0	0	0	0
4	Biodiesel production facility	0	0	0	1	0	0	0	1	0	1	1	0
5	Supply of used cooking oil	0	1	0	1	1	0	0	0	0	0	0	0
6	Production Technology for biodiesel	0	1	1	1	0	1	0	0	0	0	0	0
7	Belief in environment concern	1	1	1	1	1	1	1	0	1	1	1	1
8	Availability of technical expertise	1	1	0	0	1	1	0	1	0	1	1	0
9	Financial assistant to biodiesel producer	1	1	1	1	1	0	0	1	1	1	1	0
10	Quality of human resources	0	1	1	0	0	1	0	0	0	1	1	0
11	Biodiesel supportive vehicles	0	0	0	0	0	1	0	0	0	0	1	0
12	Government policy and support	1	1	1	1	1	1	1	1	1	1	1	1

The initial reachability matrix (IRM) was prepared based on the substitution concept.

After constructing the IRM, final reachability matrix (FRM) was created by introducing categorization. The FRM was generated by the transitivity consideration and the conversation principle as shown in Table 4. With the experts opinions FRM prepared is in compliance with step V.

**Table 4:** Final reachability matrix

	Enablers		**1**	**2**	**3**	**4**	**5**	**6**	**7**	**8**	**9**	**10**	**11**	**12**
1		Awareness to used cooking oil as biodiesel	1	1	1	1	1	1	0	1	0	1	1	0
2		Human health concern	0	1	0	1	0	0	0	1	0	1	1	0
3		Used cooking oil collection center	0	1	1	1	1	0	0	1	0	1	1	0
4		Biodiesel production facility	1	1	1	1	1	1	0	1	0	1	1	0
5		Supply of used cooking oil	0	1	0	1	1	0	0	1	0	1	1	0
6		Production Technology for biodiesel	0	1	1	1	1	1	0	1	0	1	1	0
7		Belief in environment concern	1	1	1	1	1	1	1	1	1	1	1	1
8		Availability of technical expertise	1	1	1	1	1	1	0	1	0	1	1	0
9		Financial assistant to biodiesel producer	1	1	1	1	1	1	0	1	1	1	1	0
10		Quality of human resources	0	1	1	1	1	1	0	0	0	1	1	0
11		Biodiesel supportive vehicles	0	1	1	1	0	1	0	0	0	0	1	0
12		Government policy and support	1	1	1	1	1	1	1	1	1	1	1	1

#### Level partitions

For each element, the usability set and context sets were extracted from the FRM. The functionality set consists of the factor itself and another factor to which it can impact or add, while the context set consists of the factor itself and another factor that can influence or add to it. In this stage, using an FRM, accessibility R and antecedent A sets were created. The R is composed of element I itself and other elements that could be affected (row elements), while the A consisted of element I and elements that could be affected (column elements). The intersection of R and A, i.e., R ∩ A, was then determined. The popular elements in both R and A were included. A comparison of R and R ∩ A columns was made. In the hierarchy, the element for which R and R ∩ A are equal was the upper-most element, and level I was applied to the element. The elements of level I was the elements that would be at the top of the ladder that would not go above their level towards other elements. The intersection of these sets was consequently derived for all the variables and the various factor levels shown in Table 5 were prepared. The top level of the ISM hierarchy is filled by the variables with which the usability and the intersection sets are the same. The top-level variables are those variables that in the hierarchy would not push the other variables above their level.

**Table 5:** Partitions for enablers

Enablers	Reachability	Antecedent	Intersection	Level
1	1, 7	1, 7, 9, 12	1, 7	**V**
2	2, 8, 10, 11	1, 2, 3, 4, 5, 6, 7, 8, 9, 10, 11, 12	2, 8, 10, 11	**I**
3	3, 8	1, 3, 6, 7, 8, 9, 12	3, 8	**III**
4	1, 2, 3, 4, 5, 6, 8, 10, 11	1, 2, 3, 4, 5, 6, 7, 8, 9, 10, 11, 12	1, 2, 3, 4, 5, 6, 8, 10, 11	**I**
5	5, 8, 10	1, 3, 5, 6, 7, 8, 9, 10, 12	5, 8, 10	**II**
6	6	1, 6, 7, 9, 12	**6**	**IV**
7	7, 12	7, 12	7, 12	**VII**
8	1, 3, 6, 8, 12	1, 3, 6, 7, 8, 9, 12	1, 3, 6, 8, 12	**III**
9	9	7, 9, 12	9	**VI**
10	3, 5, 6, 10	1, 3, 5, 6, 7, 8, 9, 10, 12	3, 5, 6, 10	**II**
11	2, 3, 4, 6, 11	1, 2, 3, 4, 5, 6, 7, 8, 9, 10, 11, 12	2, 3, 4, 6, 11	**I**
12	7, 12	7, 12	7, 12	**VII**

### Formation of ISM model

**Fig. 2:**
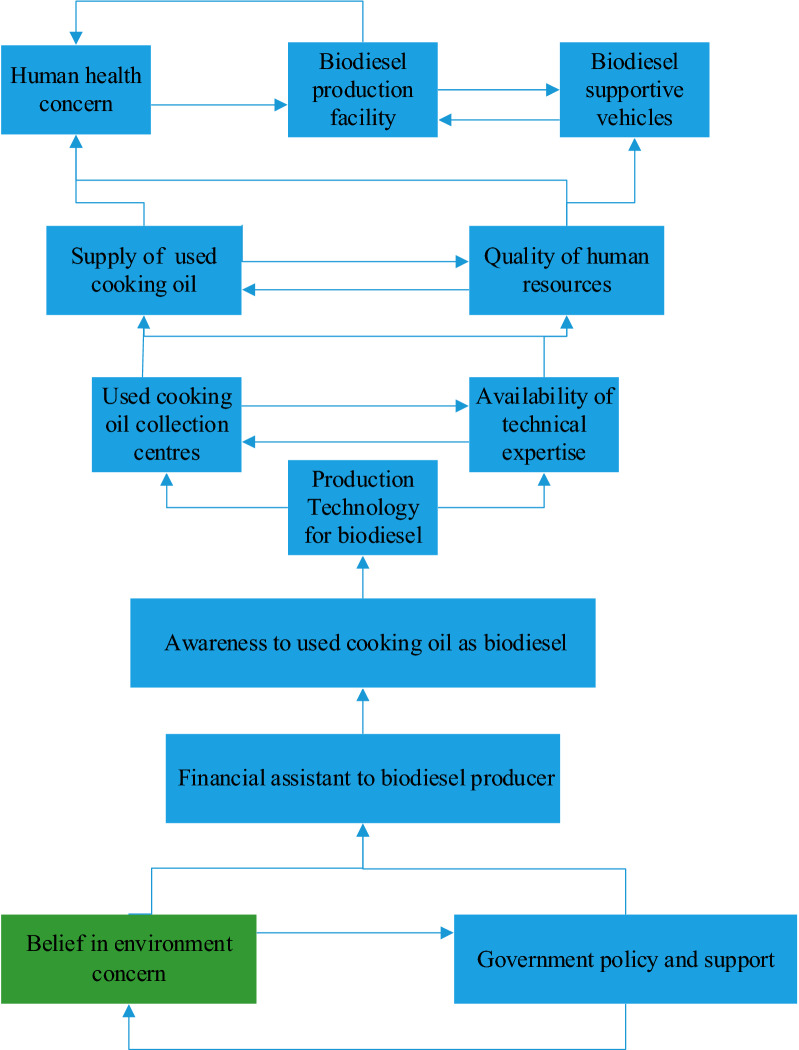
Model of ISM enablers for WCO development of biodiesel

The diagraph was then eventually translated into the ISM model after eliminating the transitivity. By using the final reachability matrix, the ISM model was created as shown in Fig. 2.

### Analysis of data using MICMAC

**Fig. 3:**
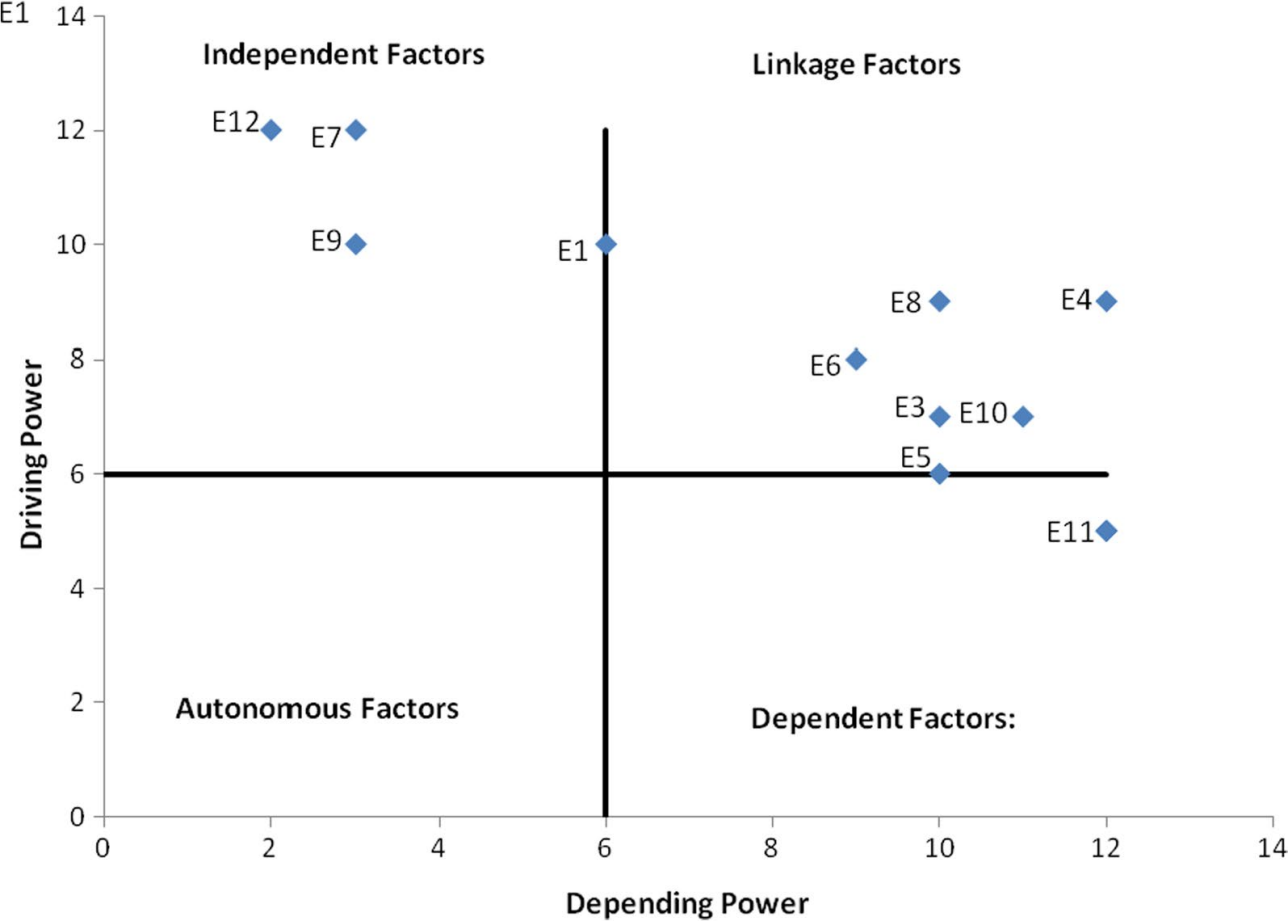
MICMAC chart

MICMAC analysis aims to analyze variables’ driving power and dependence power. The principle of MICMAC is based on the proliferation characteristics of matrixes [45]. In different categories, it is analyzed to evaluate the essential factors that lead the system. The motivating reason is estimated by summarizing the entries of interaction possibilities in the rows and the vulnerability is measured by summarizing the entries of interaction possibilities in the columns as in Table 6. The driving power of any enabler is the impact of that enabler on other remaining enablers and the dependence power is, the impact of remaining enablers on an enabler. The variables (enablers) were classified into four categories based on their drive power and dependency power, as shown in Fig. 3.

**Table 6:** Driving power and depending power

	Enablers	**1**	**2**	**3**	**4**	**5**	**6**	**7**	**8**	**9**	**10**	**11**	**12**	Driving power
**E1**	Awareness to used cooking oil as biodiesel	1	1	1	1	1	1	0	1	0	1	1	0	9
**E2**	Human health concern	0	1	0	1	0	0	0	1	0	1	1	0	5
**E3**	Used cooking oil collection center	0	1	1	1	1	0	0	1	0	1	1	0	7
**E4**	Biodiesel production facility	1	1	1	1	1	1	0	1	0	1	1	0	9
**E5**	Supply of used cooking oil	0	1	0	1	1	0	0	1	0	1	1	0	6
**E6**	Production Technology for biodiesel	0	1	1	1	1	1	0	1	0	1	1	0	8
**E7**	Belief in environment concern	1	1	1	1	1	1	1	1	1	1	1	1	12
**E8**	Availability of technical expertise	1	1	1	1	1	1	0	1	0	1	1	0	9
**E9**	Financial assistant to biodiesel producer	1	1	1	1	1	1	0	1	1	1	1	0	10
**E10**	Quality of human resources	0	1	1	1	1	1	0	0	0	1	1	0	7
**E11**	Biodiesel supportive vehicles	0	1	1	1	0	1	0	0	0	0	1	0	5
**E12**	Government policy and support	1	1	1	1	1	1	1	1	1	1	1	1	12
	Depending power	6	12	10	12	10	9	2	10	3	11	12	2	99

*Autonomous factors* These variables have poor drive power and the weak power of dependency. They are relatively disconnected from the model that they have few connections with, which can be very strong.

*Linkage factors* These variables have strong drive power as well as strong power of dependence. The fact that any action on these variables will affect others and also a feedback effect on themselves makes these variables unstable.

*Dependent variables* These variables have weak drive power but strong power of dependence.

*Independent variables* These variables have strong drive power but weak power of dependence. A factor called the ‘key factor’ with a very strong drive power falls into the category of independent or linkage factors.

It was acknowledged that it has high driving power from belief in environmental concern and government policy and support, indicating that the impact of government policies influences other enabling factors in WCO productivity. The Enablers, Government Policy and Support, and Belief in environment concern the highest driving power, meaning that enablers are independent variables responsible for supporting other enablers for WCO biodiesel development as shown in Fig. 3. Enablers, used cooking oil collection center, biodiesel production facility, supply of used cooking oil, production technology for biodiesel, availability of technical expertise and quality of human resources with high driving power and dependence power are linkage variables. Enabler biodiesel supportive vehicles having strong dependence power strongly depends upon other enablers flows under dependent variables.

## Results and discussion

Biodiesel manufacturing from used cooking oil is vibrant business potential for any country, with significant economic and environmental advantages. Vital enablers leading to the ignition and sustainable production of WCO biodiesel fuel were identified in the present research approach through a comprehensive investigation. After defining significant enablers in the production of WCO biodiesel, they analyzed it separately. Organizations cannot initiate and promote the effective production of WCO biodiesel in their operations and processes without studying these identified enablers. These prominent agents have been scrutinized further after interacting with industry and research specialists. Following the findings of this study, the barrier to the long-term deployment of WCO biodiesel is removed as a first step toward establishing or extending it as a feasible approach for WCO biodiesel production. Some of the main outcomes of this study are discussed below:

- Government policy and environmental support as well as belief in the production of biodiesel has been identified as driving mass production of WCO biodiesel.
- The application of sustainability policies is influenced by the environmental awareness and procedures of actual and potential stakeholders, along with the government. It has been indicated that despite the biodiesel generation through waste cooking oil, the level of environmental safety implementation is high because of their little awareness of the advantages of internal combustion engine oil, water, and exhaust emissions. This is consistent with the results of earlier researches which has shown that environmental awareness and environmental practices have a positive relationship [57, 59].
- Government support, in many developing economies, its considerable influence has been indicated, especially in terms of technical support. Moreover, owing to the absence of government subsidies to encourage WCO biodiesel, many restaurants, hotels, and the food industry are not inspired to supply WCO [58].
- The outcome of the MICMAC analysis shows that autonomous enablers do not exist. The lack of such enablers in the present study confirms that an important role is played by all the enablers identified. The existence of any of the enablers identified in this study can therefore act as an important obstacle to the production and promotion of biodiesel from WCO.

The results of this analysis demonstrate that some of the unavoidable leading enablers that hinder WCO biodiesel are related to the human end. This category includes ‘Availability of technical expertise, and ‘Quality of human resources’. It is acknowledged that trained experts are a vital agent in creating a vibrant ecosystem that enables WCO biodiesel to be adopted and developed by society. Moreover, current research has clarified the lack of involvement of human resources in the utilization of WCO as a key source for fuel, that not lead to improvement in the wealth of any country and decreases the realization of opportunities for more sustainable benefits [52].

## Conclusions

This research article provides the solution to the obstacle to the effective integration and implementation of WCO development factors and develops their contextual relationship to better identify the complex consequences on the development of the ISM approach of certain contributing factors. To do this, a comprehensive analysis of the intellectual literature was carried out as a first statistical step to designate the enabler factors that can influence society to the efficient implementation and commercial production of WCO biodiesel in India. Twelve enablers were recognized and then evaluated to identify the contextual re-production of WCO biodiesel and all enablers modeled using the ISM method. The ISM method is an appropriate technique for identifying the link between various enablers for WCO biodiesel production. Modeling of enablers allows biodiesel producers to better understand the interdependence and linkages of multiple enablers, resulting in more sustainable WCO usage. The allowances were categorized into linked, independent, and dependent factors using a MICMAC analysis. As a result, six enablers have been classified as ‘linkage’, one enabler has been determined as a ‘dependent’ factor while no ‘driver’ or ‘independent’ has been identified. Because the MICMAC research revealed all identified enablers play an important role, and can thus act as a combined light source for WCO’s biodiesel production and implementation.

This study extends our knowledge to include:

- Defining as a source of biodiesel, the suppliers that promote and remove obstacles in the WCO;
- To provide detailed and deeper insights upon these enablers by establishing contextual interconnection and specific impacts on the use of WCO;
- Helping one to grasp the scope for use of WCO and the industrial production of WCO-based biodiesel. ‘Linkage’ and one enabler’s biodiesel supportive vehicles have been determined to be ‘dependent’.

## Data Availability

All data generated or analyzed during this study are included in this published article.
